# Supplementary material for: Virtual Screening and Validation of Affinity DNA Functional Ligands for IgG Fc Segment
Source: Int J Mol Sci. 2024 Aug 9;25(16):8681. doi: 10.3390/ijms25168681 (PMC11354668; doi:10.3390/ijms25168681)
Supplement: Supplementary file 1 [file ijms-25-08681-s001.zip › Table S1.pdf]

Table S1 140 DNAFLs with determined 3D structures selected from PDB.

	**PDB code**	**Nucleotide sequences (5' to 3')**	**Length**	**Mode**	**ZDOCK Score**
1	3HXQ	GGCGTGCAGTGCCTTCGGCCGTGCGGTGCCTCCGTCACGCC	41	1	32.78
2	5XRZ	TTTTTTTTTTTTTTTTTCCCTTTTTTTTTTTTTTTTTTTT	40	1	29.54
3	3A5U	CCCCCCCCCCCCCCCCCCCCCCCCCCCCCCC	31	1	29.08
4	7QB3	CGGCCGTCGAAGACCCGCCAAGTGGCCG	28	1	29.96
5	3ZH2	CTGGGCGGTAGAACCATAGTGACCCAG	27	1	27.48
6	6GN7	CGCCTAGGTTGGGTAGGGTGGTGGCG	26	1	26.78
7	4GNX	TTTTTTTTTTTTTTTTTTTTTTTTT	25	1	26.24
8	8SH0	CCAGCAGGGGTTAGGGTTAG	20	1	30.22
9	7JSG	TTTTTTTTTTTTTTTTT	17	1	23.92
10	7LCC	TCCTATATAATAAAAGA	17	1	25.94
11	6SKO	TTTTTTTTTTTTTTTT	16	1	23.54
12	7XHD	GGGGTGGGTGGTGGGT	16	1	24.42
13	2C62	TTTTTTTTTTTTTTTG	16	1	25.12
14	2CCZ	TTTTTTTTTTTTTTT	15	1	25.38
15	1RDE	GGTTGGTGTGGTTGG	15	1	25.58
16	7JSH	CGCTCGCTCGCTCGC	15	1	24.18
17	5V9X	GACCTGCTGCGGATT	15	1	24.2
18	6CRM	GGGTCGGTGCCTTA	14	1	25.04
19	4JRQ	AAAAAAAAAAAAA	13	1	25.76
20	1PH1-D	GGGGTTTTGGGGT	13	1	24.1
21	1OTC	GGGGTTTTGGGG	12	1	23.9
22	4JS5	TTTTTTTTTTTT	12	1	23.14
23	7Y01	CCCCCCCCCCCC	12	1	20.2
24	3UGO	TGTACAATGGG	11	1	22.5
25	5XS0-Y	CCCCCCCCCC	10	1	20.34
26	6WDZ	TAGTATTACC	10	1	24.16
27	4OU6	TTTTTTTTTT	10	1	21.98
28	5N9D	GGGTTAGGGT	10	1	22.44
29	6CQ2	TTCCGCTTGA	10	1	21.38
30	2A0I	TGGGGTGTGG	10	1	22.46
31	2KN7	CAGTGGCTGA	10	1	22.26
32	4HIM	GGATACGGT	9	1	21.82
33	6BUX	AATCCCAAA	9	1	20.3
34	6KBS	GGTCGATTC	9	1	22.2
35	7JSI	TTTTTTTT	8	1	19.68
36	7CRE	TCAGCCTC	8	1	20.26
37	5FGP	GCGGCGG	7	1	20.42
38	2O5C	CGCAACT	7	1	20.4
39	4POG-X	TTTTTTT	7	1	19.42
40	6JVY	TGTGTGT	7	1	19.04
41	1MJE	TTTTTT	6	1	18.4

42	6S3M	TTTTTG	6	1	18.6
43	3B39	AAAGCC	6	1	18.88
44	5O6E	TTTGGT	6	1	18.98
45	5O6B	GGGTTT	6	1	19.26
46	1ZZI	CTCCCC	6	1	19.3
47	1QZG	GGTTA	5	1	17.08
48	1XHZ	TTTTT	5	1	17.36
49	4I28	AAAAA	5	1	17.18
50	3HXO	GGCGTGCAGTGCCTTCGGCCGTGCGGTGCCTCCGTCACGC	40	2	28.96
51	6U82	GCTAATCTAATCAACCGCAGGTTGATTAGCCCATTAGC	38	2	29.9
52	5HRU	TCGATTGGATTGTGCCGGAAGTGCTGGCTCGA	32	2	30.48
53	5CMX	TGACGTAGGTTGGTGTGGTTGGGGCGTCAC	30	2	28.48
54	5HTO-F	CGATTGGATTGTGCCGGAAGTGCTGGCTCG	30	2	27.02
55	7NTU	GTCCGTGGTAGGGCAGGTTGGGGTGAC	27	2	30.1
56	5EW1	TCCGTGGTAGGGCAGGTTGGGGTGAC	26	2	26.14
57	7V5N	GCGGTTGGTGGTAGTTACGTTCGC	24	2	28.34
58	2L5K	CAGTTGATCCTTTGGATACCCTG	23	2	27.22
59	4PCB	GCACCGAAGGTGCGTATTCTTG	22	2	27.22
60	4KDP-H	CGCAGCGCGCA	11	2	24.2
61	3N1K	AAAAAAAAA	9	2	22.5
62	2VYE	TTTTTTTTT	9	2	22.82
63	6LMR	AACACCT	7	2	20.54
64	6QEM	TTTTTTTTTTTTTTTTTTTTTTTTTT	26	3	25.62
65	4GOP-L	TTTTTTTTTTTTTTTTTTTTTT	22	3	25.34
66	4J1J	ACCAAACAACCCACCCA	17	3	25.88
67	4QQW	AAAAAAAAAAAA	12	3	20.48
68	5N96	AGGGTTTTTT	10	3	23.94
69	5N9E	TGGGGATTT	9	3	21.66
70	5N8R	GAGCACTGC	9	3	21.98
71	5HTO-C	TTCGATTGGATTGTGCCGGAAGTGCTGGCTCGAA	34	other	28.62
72	4NOE	TTGCGCTTGCGCTTGCGCTTGCGCTTGCGC	30	other	30.2
73	7ZQS	AAAGGGGGTGTTTGTGCGGTGTGGAGTGCG	30	other	27.54
74	7YPO	AAAAAAAAAAAAAAAAAAAAAAAAAAAA	28	other	32.64
75	6LBR-C	ACGGATTTGATTAGGTATGTGG	22	other	28.64
76	7CUH	GGTTACAGGGGTTACGGT	18	other	24.86
77	7X7G	GGGGTGGGAGGTGGGT	16	other	24.9
78	4JS4	AAAAAAAAAAAAAAA	15	other	25.4
79	5EAN	ACTCTGCCAAGAGGA	15	other	23.1
80	4ESV	TTTTTTTTTTTTTT	14	other	24.24
81	1UUT	CAGCTCTTGAGCTG	14	other	23.24
82	5F55	GATGTACGCTAGGC	14	other	26.16
83	7T23	TTTTTTTTTTTTT	13	other	22.66
84	7AUD	GTACCCGATGTGT	13	other	23.64
85	4RUL	TATGCGATTTGGG	13	other	23.34

86	1PH4-D	GGGGTTTTGGCG	12	other	28.62
87	7WM3	TTAGGGTTAGGG	12	other	25.94
88	1PA6	GGGGTTTTGAGG	12	other	28.16
89	4HJA	ACGGTTACGGT	11	other	23.2
90	7YPQ	AAAAAAAAAAA	11	other	22.12
91	7XF1	TTTTTTTTTTT	11	other	21.28
92	4KI2-D	TGTACAAATGG	11	other	23.94
93	1PH8-D	GGGTTTTGCGG	11	other	26.34
94	1PH9-D	GGGTTTTGAGG	11	other	25.78
95	3KJP	GTTAGGGTTAG	11	other	24.22
96	4HJ9	CGGTTACGGT	10	other	23.42
97	1XJV	TTAGGGTTAG	10	other	22.42
98	6WE0	TAATATTACC	10	other	20.08
99	7KIJ	TATTATTACC	10	other	21.08
100	2FR4	CTGCCTTCAG	10	other	22.46
101	5N9F	GGGGACGATC	10	other	23.2
102	5ZVA	ATTTTCAACT	10	other	23.14
103	4KI2-E	GTACAATGGG	10	other	24.76
104	4KDP-J	CGCGCAGCGC	10	other	22.66
105	4HIK	GGTTACGGT	9	other	21.74
106	4HID	GCTTACGGT	9	other	20.62
107	4HIO	GGTAACGGT	9	other	22.14
108	4HJ5	GGTTTCGGT	9	other	22.18
109	4HJ7	GGTTAGGGT	9	other	21.98
110	4HJ8	GGTTACGCT	9	other	22.28
111	7TZV	TAGTCTACT	9	other	23.38
112	1PH2	GGGGTTTTG	9	other	21.2
113	5N8Z	CTCTCCCTT	9	other	20.6
114	5ZVB	ATTTTCAAT	9	other	22.3
115	5N90	TTGTGGTGT	9	other	21.2
116	5N98	TAGGGTTTT	9	other	20.48
117	5XS0-Z	CCCCCCCCC	9	other	19.2
118	5N8U	CTCTCCCT	8	other	18.92
119	6WE1	AATATTAC	8	other	21.28
120	1I7D	CGCAACTT	8	other	21.2
121	5XJZ	GCACGCGC	8	other	22.42
122	1JMC	CCCCCCCC	8	other	20.02
123	1ZZJ	CCCCTCCC	8	other	20.3
124	5FHD-C	TTTTTCCG	8	other	20.32
125	8DWJ	TCGGCTG	7	other	19.92
126	7LW9	TGAAGGG	7	other	21.02
127	4TYN	AAAAAAA	7	other	19.58
128	1KIX	TTTGGGG	7	other	18.16
129	1MW8	CTTCGGG	7	other	18.62

130	5FHD-E	TTTTTTC	7	other	19.22
131	6XB8	GCTCTT	6	other	20.34
132	8GBJ	CCCCCC	6	other	17.66
133	7BJQ	CATGGC	6	other	18.08
134	5SWW	ATCGGG	6	other	17.96
135	3NH1-F	ATAGG	5	other	18.58
136	7OMY	ATGTC	5	other	18.48
137	8BAR	AAGAC	5	other	17.28
138	7ZES	CGCGT	5	other	15.96
139	1J5K	TCCCT	5	other	16
140	7CSZ	GACGG	5	other	19.08

Notes: The table is categorized based on distinct binding sites. No.1 - No.49 represents the DNAFLs that clearly binds to the Fc segment in mode 1, followed by the listing of DNAFLs belonging to mode 2 and mode 3, respectively. "Other" indicates complete localization of the entire DNAFL within the Fab segment, along with some unfavorable cases observed in the docking results.
